# Does tinnitus lead to chaos?

**DOI:** 10.1016/j.bjorl.2020.11.022

**Published:** 2021-01-15

**Authors:** Maryam Sadeghijam, Abdollah Moossavi, Mahdi Akbari

**Affiliations:** a Iran University of Medical Science, School of Rehabilitation, Department of Audiology, Tehran, Iran; b Iran University of Medical Science, Department of Otolaryngology and Head and Neck Surgery, Tehran, Iran; c Iran University of Medical Science, School of Rehabilitation, Department of Audiology, Tehran, Iran

Tinnitus, the perception of sound in the ears or the head without an actual external origin, is an influential problem in public health, negatively affecting the life of about 15% of the world’s adult population.^1^ Considering the prevalence of this disorder, and the need for reliable treatment, much research has been done to identify the mechanisms and eventual management strategies for tinnitus. While some articles consider the deafferentation of the auditory bottom-up tract with or without hearing loss as the main culprit, others focus on the deficiency of the top-down noise cancelling mechanism or even both factors simultaneously.^2^ The neural deafferentation in the central auditory structures resulting from cochlear damage makes changes in the central auditory tract including tonotopic map reorganization, hyperactivities in auditory cortex and thalamus and increased neural synchronization, especially in areas related to the auditory cortex that are affected by hearing loss.^2^ It seems that as a result of any decrease in auditory inputs and imbalance between excitation and inhibition, a compensatory mechanism is activated resulting in increased neural spontaneous activity and synchronization.^3^ Although increased activities in the auditory brain areas and tonotopic map reorganization seem to be the main reasons for tinnitus, it cannot always be the case. Since tinnitus is not present during sleep conditions, some brain areas related to cognition must be involved in the perception of tinnitus. For a conscious perception of acoustic stimuli, in addition to the activity of the main auditory center in the brain, other centers such as parts of the prefrontal lobe, parietal lobe, cingulate and insula must be activated. These structures comprise two main networks: the perception network including anterior and posterior cingulate cortex and some parts of parietal and frontal lobes, and Precuneus and the salience network covering anterior and posterior parts cingulate cortex and anterior insula. Some variations in the activity of these networks has been proven by electroencephalogram/ magnetoencephalogram investigations in tinnitus patients.^1^, ^2^, ^3^ The thalamocortical dysrhythmia theory states that the low frequency brain waves such as theta and delta increase due to the auditory deafferentation and high frequency brain waves i.e. gamma band increase as a result of the reduced inhibition from the thalamus to cortex.^2^

The same studies indicate the involvement of more areas of the brain including the parahippocampus, hippocampus as well as amygdala, which are parts of the learning network.^3^ On the other hand, distress has been proven to be another reason for tinnitus, increasing the likelihood of tinnitus when combined with hearing loss. Thus, it appears that the generation and perception of tinnitus can be resulted from the activation of a global network including perception network, salience network, learning network and distress network.^1^, ^2^, ^3^

As a conclusion, the network theory could be a perfect explanation for tinnitus generation. At first, it was assumed that the networks would be completely coincidental i.e., the node regions in each network were randomly connected and all nodes had the same importance in each network.^3^ Later on, the scale-free networks were presented. In these networks, there are more obvious interconnections among the nodes, which in turn increases the potential of the whole unit. In the scale-free network theory, any harm to the major hubs would result in the general deficiency and harm to the entire network.^3^ Today, it has become clear that the brain does not follow a single network law, but multiple networks are simultaneously activated based on the number of its functions and stimuli.^3^ Therefore, since multiple scale-free networks are probably activated simultaneously in tinnitus, the management approaches by acoustical and electrical neuromodulation must have been effective because major hubs can be disrupted or affected.^3^, ^4^ However, researches have shown that this type of tinnitus management has had only a temporary and limited effect for a small percentage of patients,^4^ showing inefficiency of network model. So, it appears that a new mechanism for tinnitus might be considered.

Different studies have shown that the human brain is not a linear system, but rather a dynamic and nonlinear one.^5^ Chaos is a phenomenon concerning nonlinear systems.^5^ According to chaos theory, in a deterministic nonlinear and dynamic system that has apparent irregularity and random states, a small change in the input can bring about major changes in the output, because of the presence of underlying patterns, interconnectedness, constant feedback loops, repetition, self-similarity, fractals, and self-organization in this system.^5^ Any small change in the human auditory system like a very small auditory deafferentation can produce widespread and diffused changes in many areas of the brain and manifest annoying tinnitus. It seems that the brain is an attractive combination of regularity and chaos; the combination that is highly sensitive to its initial conditions and any small change in the initial state can result in some large alterations in the latter state in the whole brain. The brain as a whole has unpredictable behavior and shows irregularity, but if we look at it in detail, we see a complex of nonlinear and linear equations that work together.^5^ Therefore, according to butterfly effect in chaos theory, any small change in the hearing system leads to annoying tinnitus which causes drastic changes in the whole system.^5^

There are several articles concerning the brain wave alterations in tinnitus patients compared to normal subjects and their results have not always coincided.^1^ For example, Weisz et al. have stated that alpha frequency band decreases and theta and gamma frequency bands increase in the tinnitus patients, while Lee E van der et al. just pointed to an increase in gamma band in the contralateral auditory cortex. On the other hand, Adjamian et al. suggested that an increasing of the gamma band is not related to tinnitus and increasing of the delta band is a sign of tinnitus.^1^ The locations of these changes also have not always been the same.^1^ Regardless of the homogeneity of participants, some researchers have found an increasing power of the brain rhythms in the auditory cortex area, and others have realized more widespread areas of the brain being affected.^1^ These differences are probably the outcome of the nonlinear nature of the brain waves. Besides, most EEG studies have pointed out to an increment of power of the frequency rhythms (such as delta and theta).^1^ The increased power of a rhythm means the creation of a small regularity in the irregular (chaotic) brain which probably causes a more tremendous irregularity and a vigorous reaction in the entire brain leading to annoyance that may develop the tinnitus. It is noteworthy that some researchers, through investigating the brain behavior, have found that the brain functions follow the chaos theory.^5^

So, regarding the nonlinear and dynamic structure and function of the brain, it is acceptable that a small change in the brain input can bring about some huge and irregular changes in the overall brain function. On the other hand, given the concept of tinnitus and the evaluation of different brain changes in this phenomenon and also the inefficiencies of the current management methods mostly based on the current theories, it seems that chaos theory in the generation of tinnitus and its complications can resolve the present contradictions and complete previous theories. Surely, more research is needed to confirm and validate this hypothesis.

## Conflicts of interest

The authors declare no conflicts of interest.
